# *Candida albicans*/*Staphylococcu*s *aureus* Dual-Species Biofilm as a Target for the Combination of Essential Oils and Fluconazole or Mupirocin

**DOI:** 10.1007/s11046-017-0192-y

**Published:** 2017-08-19

**Authors:** Aleksandra Budzyńska, Sylwia Różalska, Beata Sadowska, Barbara Różalska

**Affiliations:** 1 Laboratory of Microbiological and Technical Services, University of Lodz, Banacha 12/16, 90-237 Lodz, Poland; 2 Department of Industrial Microbiology and Biotechnology, University of Lodz, Banacha 12/16, 90-237 Lodz, Poland; 3 Department of Immunology and Infectious Biology, Faculty of Biology and Environmental Protection, Institute of Microbiology, Biotechnology and Immunology, University of Lodz, Banacha 12/16, 90-237 Lodz, Poland

**Keywords:** Dual-species bacterial/fungal biofilm, Essential oils

## Abstract

The effectiveness of essential oils (EOs), fluconazole (FLU) and mupirocin (MUP) used alone or in combination against mono-species and mixed *Candida albicans/Staphylococcus aureus* biofilms was examined. An experimentally established dual-species biofilm model, verified by fluorescence microscopy and viable cell counting, was used. Selected commercial EOs were tested: geranium, citronella and clove oils, which have been chemically characterized and found to differ in the content of the main components (qualitative and quantitative). As expected, *C. albicans* and *S. aureus* biofilms were less susceptible to fluconazole and mupirocin action, respectively, compared to the planktonic counterparts. However, the drug effectiveness in combination with the EOs was significantly improved, giving enhancement of biofilm eradication than caused by the antibiotics alone. Moreover, dual-species biofilm formation was limited by sub-MIC of EOs, and preformed mixed biofilm was eliminated more efficiently by combined action of drugs and EOs.

## Introduction

*Candida albicans* in specific host-dependent conditions is an effective opportunistic organism. Invasive candidiasis usually results from yeast-to-hyphae transformation and tissue colonization due to the formation of a biofilm [1]. The possibility of the development of polymicrobial biofilms, consisting of both fungi and bacteria, should also be considered in pathogenesis of various infections. *C. albicans* is able to grow together with *Staphylococcus aureus*, *S. epidermidis* and *Enterococcus* sp. in the course of blood-borne infections, with *Gardnerella vaginalis* during vaginal infections, *Pseudomonas aeruginosa* in cystic fibrosis and diverse other bacteria or fungi in oral as well as in skin/wound infections [2–6].

Since it is known that *C. albicans* isolates resistant to azoles emerge often, several approaches to overcome this have been proposed. The most convincing is using a combination of fluconazole and various classes of non-antifungal agents [8, 9]. Similarly, successful treatment of drug-resistant *S. aureus* is a major challenge for medicine. The reports on the increasing resistance to pseudomonic acid (mupirocin)—one of the most potent topical antibiotics used against MSSA and MRSA—are of serious concern and also necessitate the search for alternatives [10].

Interesting strategies of the treatment are derived from the experience of ethnomedicine inspired by the use of natural-origin products. Our laboratory has investigated antimicrobial activity of plant essential oils, which became the basis for the present study [11].

## Materials and Methods

### Research Material

- Suspensions of *C. albicans* ATCC 10231 cultured in RPMI-1640 medium with 2% glucose and *S. aureus* NCTC 8325-4 in TSB with 0.25% glucose.
- Geranium, citronella and clove oils (EOs) from Pollena Aroma, Poland; fluconazole (FLU) and mupirocin (MUP) from Sigma, USA.

### Minimal Inhibitory Concentration (MIC)

The MICs of the EOs tested at a concentration range of 0.48–7.8 µl ml^−1^ were determined by a broth microdilution method according to the EUCAST guidelines [12] with minor modifications [11]. The same method was used to assess MICs of drugs: The concentration range was fluconazole—0.25–8.0 µg ml^−1^ and mupirocin—0.0625–8.0 µg ml^−1^.

### Minimal Biofilm Eradication Concentrations (MBEC)

The MBEC of EOs, FLU, MUP used separately or in combination were determined against *C. albicans* and *S. aureus* 24-h-old biofilms formed in the 96-well microplates. Biofilms were treated for the next 24 h with EOs (range MIC—16 × MIC). In other set of experiments, the *C. albicans* biofilm was exposed to fluconazole (range MIC—512 × MIC), while the *S. aureus* biofilm was exposed to mupirocin (range MIC—16 × MIC). The MBEC, decreasing metabolic activity of biomass by 50 and 80% (MBEC_50_; MBEC_80_), were evaluated using XTT reduction assay, as recommended by the manufacturer. The combined effect of FLU or MUP with EOs was assessed in a separate experiment, by applying each agent at a predetermined MBEC_50_.

### Dual-Species Biofilm Eradication

- Evaluation of fungi and bacteria participation in mixed biofilm, developed on the surface of round glass slides (area of 19.6 mm^2^) placed in the wells of 96-well microtiter plates, was performed. Three variants of suspension (100 µl) application (1 × 10^6^ CFU/ml of yeast or 1 × 10^7^ CFU/ml of bacteria) were performed: (A) yeast 2 h before bacteria; (B) bacteria 2 h before yeast; and (C) yeast and bacteria simultaneously. A quantitative evaluation was performed after 24-h incubation at 37 °C. Slides were removed, washed in PBS, transferred to the tubes with 1 ml of PBS and sonicated (5 min). Tenfold dilutions were made and cultured on SDA medium supplemented with chloramphenicol (selective for *Candida*) and Chapman medium (selective for *Staphylococcu*s). After overnight incubation at 37 °C, the colonies were counted.
- Morphology of mixed biofilms developed in a Lab-Tek II chamber slide (Nunc, Denmark) was tested after staining with fluorescent dyes: 5 µM of SYTO 9 (Molecular Probes, USA) and 0.5 ml of calcofluor white (Sigma, USA). The images were captured using AxioCam HRC camera combined with an Axiovert 200 M (Zeiss, Germany) inverted microscope equipped with a Plan-Neofluar objective (63 ×/1.25 oil).
- Dual-species preformed biofilm (24 h old) was treated with FLU, MUP applied alone or in combination with EO, each at MBEC_50_ active against mono-species biofilms. The degree of biofilm eradication was evaluated after 24-h incubation at 37 °C, using Alamar Blue reduction assay according to the manufacturer’s recommendations.

### Statistical Analysis

The results are provided as the mean ± S.D. When applicable, statistical differences between groups were evaluated using the Mann–Whitney *U* test using STATISTICA 12.0 software (StatSoft Inc., USA). *P* < 0.05 was considered significant.

## Results

Commercial EOs—geranium, citronella and clove oils, differing in content of the main components—were chosen based on the results of Budzyńska et al. [11]. Eugenol was identified in the clove oil (86.2%) but not in citronella and geranium oils. Clove oil contained also 10.4% of (E)-β-caryophyllene, which was detected in the two other oils at trace levels (0.2–0.9%). Citronella and geranium oils enclosed compounds absent in clove oil—high concentration of citronellal (36.2%) and geraniol (22.4%) in citronella oil—whereas geranium oil was rich in geraniol (10.5%) and chemically similar citronellol (44.0%). Geranium oil as the only contained also citronellyl formate (9.8%).

**Fig. 1:**
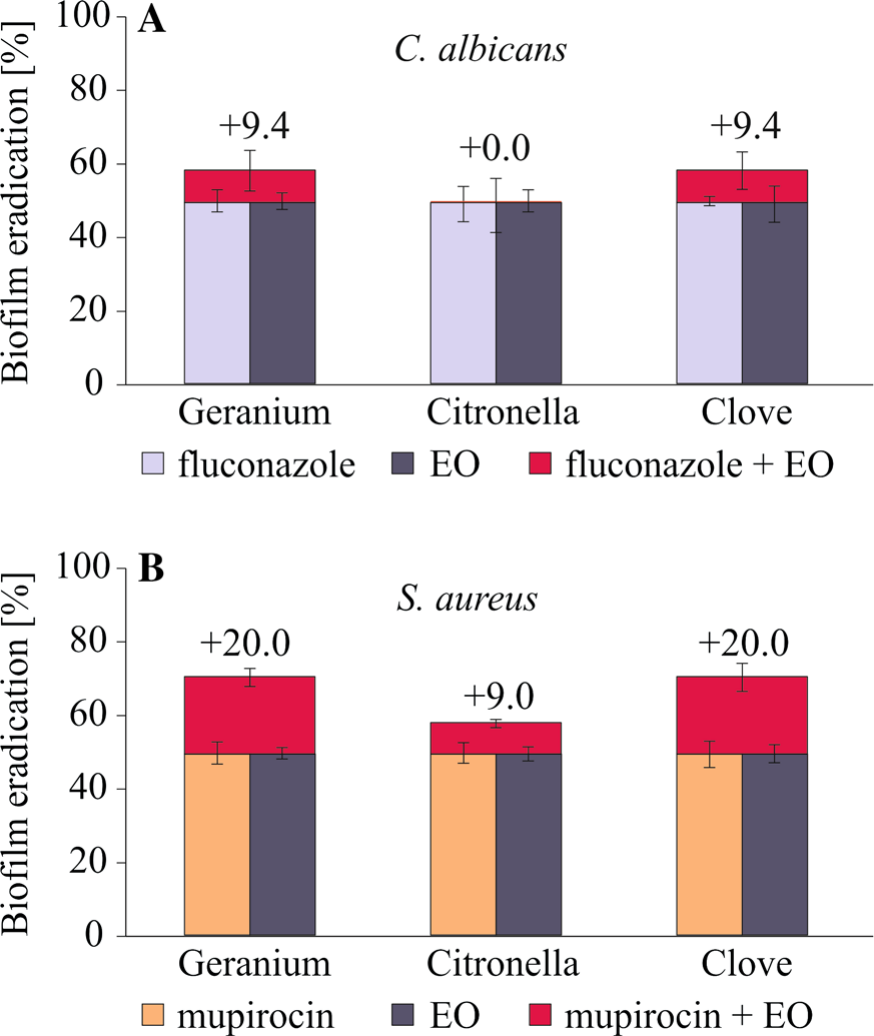
A comparison of FLU, MUP and EOs activity used separately or in combination against *C. albicans* ATCC 10231 and *S. aureus* NCTC 8325-4 preformed mono-species biofilms, assessed by XTT reduction assay. The effect was evaluated by applying each agent at MBEC_50_ for 24 h of incubation: FLU (*bright blue bars*); MUP (*orange bars*); EO (*navy blue bars*); FLU + EO or MUP + EO (*upper red boxes*; +the percentage of eradication enhancement). (Color figure online)

The first step was an evaluation of mono-species biofilm of *C. albicans* and *S. aureus* susceptibility to eradication by conventional drugs alone or in combination with the oils. The results presented in Table 1 indicate that the MBEC_50_ of EOs for *C. albicans* biomass was obtained at 2 × MIC and the MBEC_80_ value was reached at 4 × MIC. The higher concentrations of EOs were necessary to reduce *S. aureus* biofilm. When EOs were tested together with the drugs used at MBEC_50_ established when used separately, significantly stronger reduction in biomass was observed (Fig. 1).

**Table 1:** Essential oils (EOs) and chemotherapeutics (FLU, MUP) anti-biofilm activity

Oils	*C. albicans* ATCC 10231		*S. aureus* NCTC 8325-4	
	MBEC_50_	MBEC_80_	MBEC_50_	MBEC_80_
Geranium (µl ml^−1^)	1.9 (2 × MIC)	3.8 (4 × MIC)	7.6 (4 × MIC)	15.6 (8 × MIC)
Citronella (µl ml^−1^)	1.9 (2 × MIC)	3.8 (4 × MIC)	3.8 (4 × MIC)	15.6 (16 × MIC)
Clove (µl ml^−1^)	1.9 (2 × MIC)	3.8 (4 × MIC)	15.6 (4 × MIC)	31.2 (8 × MIC)
Fluconazole (µg ml^−1^)	64.0 (64 × MIC)	>512.0 (>512 × MIC)	–	–
Mupirocin (µgml^−1^)	–	–	1.0 (2 × MIC)	4.0 (8 × MIC)

The minimal concentration able to eradicate 50% (MBEC_50_) or 80% (MBEC_80_) of preformed mono-species biofilm metabolic activity, evaluated by XTT reduction assay

**Fig. 2:**
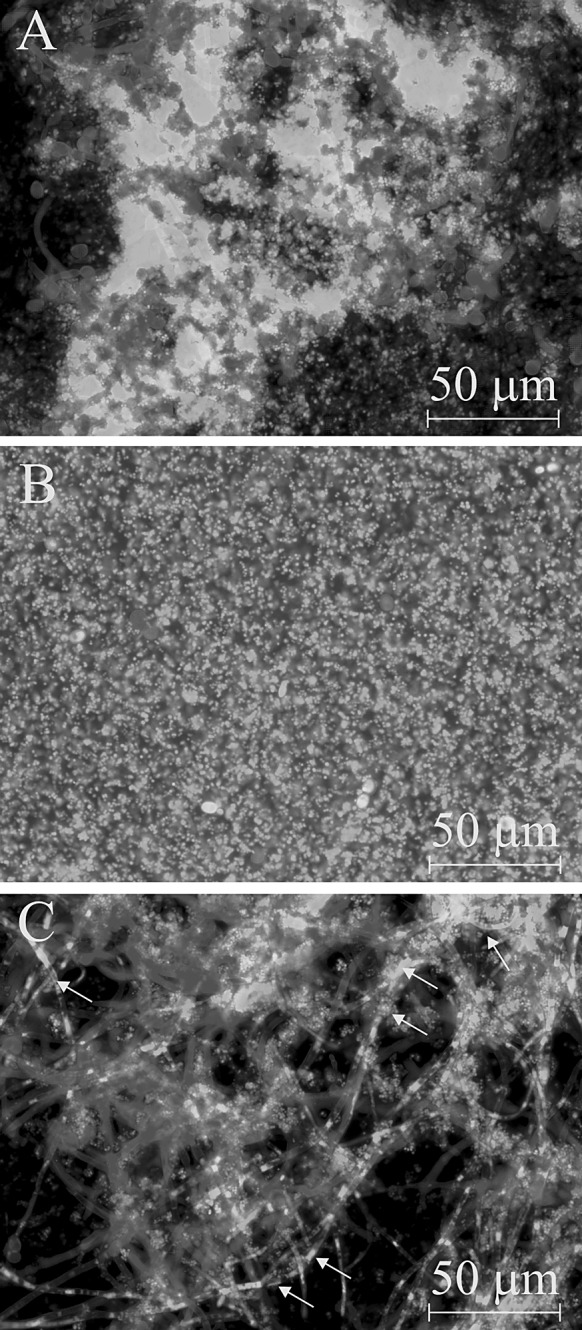
Dual-species biofilms formed in three different variants of methodology: **a** yeast applied 2 h earlier than bacteria; **b** bacteria applied 2 h earlier than yeast; **c** yeast and bacteria applied simultaneously. Biomass developed in the chambers was stained with 5 µM of SYTO 9 + 0.5 mg/mL of calcofluor white fluorescent dyes. *S. aureus* and *C. albicans* are stained *green* and *blue*, respectively. Large *S. aureus* aggregates adhered to *C. albicans* hyphae are indicated with *arrows*. The images were captured using AxioCam HRC camera combined with an Axiovert 200 M (Zeiss, Germany) inverted microscope equipped with a Plan-Neofluar objective (63 ×/1.25 Oil). (Color figure online)

Next, the effectiveness of mixed biomass destruction by combining EOs and drugs was tested. It required the establishment of optimum conditions for the dual-species biofilm model. The data were obtained by differential fluorescent staining, morphological analysis and CFU calculation. The microscopy observations indicated that the simultaneous occurrence of the contact of microbes with the surface (variant C) resulted in the formation of equally numerous dual-species populations (Fig. 2c), with the presence of different yeast morphotypes (blastospores, germ tubes, pseudohyphae and hyphae) within. Many of the staphylococcal clusters were seen to be adhered mainly to hyphae. In variant B, the formed biofilm consisted mainly of *S. aureus* microcolonies (Fig. 2b). However, when the adhesion of *C. albicans* started 2 h before *S. aureus* (variant A), fungal cell density was almost equal to *S. aureus* microcolonies, and a relatively low variability of *C. albicans* morphological forms was found (Fig. 2a). The CFU counts revealed that only simultaneous application of *C. albicans* and *S. aureus* on the surface resulted in the formation of a greater number of *C. albicans* (1.7 times higher than observed in the corresponding mono-species biofilm). In contrast, when the application of *S. aureus* preceded *C. albicans*, the biofilm consisted mainly of *S. aureus* microcolonies with the number of yeasts being 22 times lower than found in the mono-species biofilm. In all variants (A–C), the number of *S. aureus* in the mixed biofilms was up to 7.9 times higher than in the control mono-species staphylococcal biofilm.

**Fig. 3:**
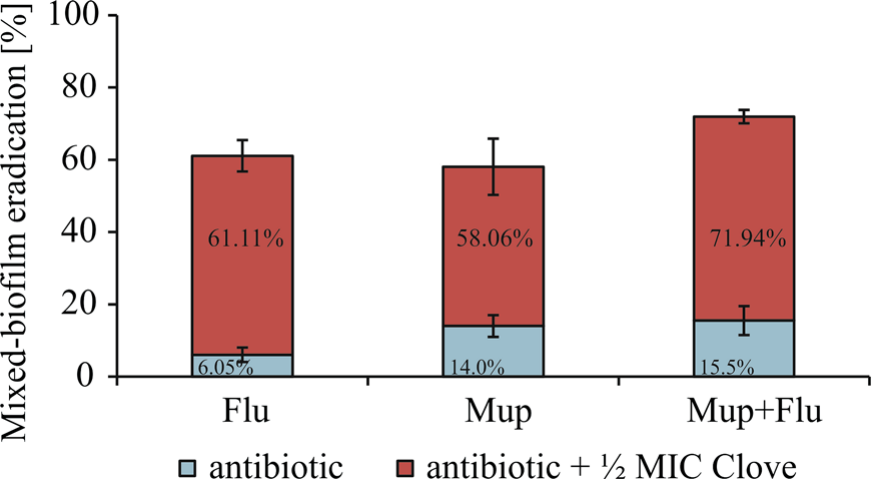
A comparison of FLU, MUP, FLU + MUP (*bottom blue bars*) activity alone and combined with clove oil (*upper red bars*) against *C. albicans* ATCC 10231 and *S. aureus* NCTC 8325-4 preformed dual-species biofilms, evaluated by Alamar Blue reduction assay. The percentages of the biofilm eradication are typed in the appropriate bars. (Color figure online)

Version C of the mixed biofilm formation model was selected for the next stage of the study. It was demonstrated that FLU or MUP in combination with clove oil (selected as the most active) against preformed mixed biofilm was very efficient. It resulted in a tenfold increase in anti-biofilm activity of fluconazole and a fourfold increase in the total efficiency of mupirocin (Fig. 3—depicted as red boxes).

## Discussion

The chemical characteristics of essential oils, performed by us previously, remain in compliance with the data published in this regard [13–15]. Noteworthy is the high content of eugenol in clove oil, citronellal and geraniol in citronella oil and citronellyl formate in geranium oil, which was absent in other EOs. Many authors [16–18] reported that eugenol and citral reduced bacterial adherence when used at sub-MIC, affected their virulence, and was not associated with any risk of resistance development. However, it remains to be determined which of the predominant citronella and geranium oil components could be responsible for their such strong activity, which in some contexts can be equal or higher than clove oil. It is important to note, however, that not always dominant constituent is responsible for the high antimicrobial potential of the essential oil in which rather synergistic activity of several components occurs.

Essential oils can be useful for medical purposes; nevertheless, they can affect both microbial and eukaryotic cells. The concentrations of EOs reported as antimicrobial are very often also cytotoxic [19–22]. Therefore, their use in topical therapy seems to be most reasonable, e.g., in the treatment of the wounds or ulcers complicated by polymicrobial infections. Interesting study in this respect has been presented by Kandimalla et al. [23] who reported anti-*Candida* and anti-inflammatory property of citronella oil in diabetic wound healing. Similar optimistic report published by Warnke et al. [24] concerns anti-bacterial activity of EOs mixture (mainly based on eucalyptus oil) in patients with incurable head and neck cancer and associated malodorous necrotic ulcers.

For many natural products, including essential oils, few mechanisms of anti-biofilm action are considered—the direct biocidal activity, inhibition of the expression of adhesins, interruption of intercellular communication and/or in the case of dimorphic fungi interference in morphogenesis [25–28]. Since both morphological forms play a role in *C. albicans* biofilm development, prevention of blastospores adhesion and their differentiation in filamentous form seems to be good therapeutic option. Such an effect of geranium, citronella and clove oil components has been previously described [17, 29] with clove oil as the most potent in this respect. Khan and Ahmad [30], similarly as in our study, reported an approximately 50% reduction in biofilm and germ tube formation of *C. albicans* by clove oil used at sub-MIC. Components of geranium and citronella oils have also been described as possessing antimicrobial activity, but precise mechanisms, other than the action on the integrity and function of the cytoplasmic membrane and various effects on microbial metabolome, remain not well known [19, 20, 30]. It is suggested that the enhanced effectiveness of conventional drugs by EOs may be attributed to so-called chemosensitization effect [8, 31–36]. However, these studies mostly concerned to mono-species biofilms or dual-species communities, but consisted of bacteria. Conversely, our report is one of the few to investigate the anti-biofilm efficiency of EOs against dual-species fungal–bacterial biofilms. A similar range of research has been published by Pekmezovic et al. [37] who demonstrated activity of pompia and grapefruit EOs against dual-species *Pseudomonas aeruginosa* and *Aspergillus fumigatus* biofilms. Harriott and Noverr [4] found that *C. albicans* + *S. aureus* biofilm exhibits an altered drug sensitivity profile compared to the monoculture: no difference in sensitivity of yeasts to amphotericin B but greatly enhanced resistance of staphylococci to vancomycin. A similar study, carried out by Li et al. [38], showed a synergistic effect of fluconazole and minocycline to combat *C. albicans* + *S. aureus* biofilm, but only against “young” mixed biofilm. In our study, the combination of FLU and MUP also did not cause much better eradication of dual-species biofilm, but the addition of EO gave a satisfactory effect. Thus, our in vitro model of dual-species biofilm is a valid target for measuring of EOs activity. In conclusion, selected EOs could be used to develop the strategies to prevent/control fluconazole-resistant yeast or mupirocin- and methicillin-resistant staphylococci.

It is now widely accepted that bacterial/fungal co-infections result in increased colonization of host tissues and greater combined virulence and resistance [2, 6, 7, 33, 39]. Study by Kong et al. [40] using mouse model of oral candidiasis showed important clinical implications of this phenomenon. The authors demonstrated that yeast infection predisposes the host to development of disseminated bacterial (*S. aureus*) disease. In further study, the same group of Kong et al. [41] found that when *S. aureus* and *C. albicans* grow together, enhanced bacteria tolerance to antimicrobial drugs occurs, mediated by fungal polysaccharides of the biofilm matrix. Similar observation has been recently published by Kean et al. [42] who showed in vitro and in vivo (*Galleria mellonella* model) that in polymicrobial biofilm other matrix component—extracellular DNA—supports *S. aureus* adhesion to yeast cells and promotes stability of the matrix. Larvae co-infection with both bacteria and fungi increased their mortality by approximately 20%, and when miconazole was used as therapeutics, loss of drug efficacy was observed.
